# Temporal Transcriptomic Profiling of NMBA-Induced Rat Esophageal Squamous Carcinogenesis Identifies Early Inflammatory Activation and Late NRF2-Associated Oxidative-Stress Remodeling

**DOI:** 10.3390/ijms27177632

**Published:** 2026-08-26

**Authors:** Ni Shi, Tong Chen

**Affiliations:** 1 Division of Medical Oncology, Department of Internal Medicine, The Ohio State University, 410 West 12th Ave., Columbus, OH 43210, USA; ni.shi@osumc.edu; 2 Comprehensive Cancer Center, The Ohio State University, Columbus, OH 43210, USA

**Keywords:** esophageal squamous cell carcinoma, *N*-nitrosomethylbenzylamine, rat model, esophageal carcinogenesis, transcriptomic profiling, inflammatory signaling, NRF2 pathway, oxidative stress

## Abstract

Temporal molecular events during early esophageal squamous carcinogenesis remain incompletely defined, in part because human precursor tissues are difficult to obtain sequentially. We used the *N*-nitrosomethylbenzylamine (NMBA)-induced rat model to characterize stage-associated transcriptional programs during esophageal squamous carcinogenesis. Vehicle-control reference esophageal tissues and NMBA-treated esophageal tissues, collected at weeks 6 and 29, were profiled using Affymetrix Rat Genome 230 2.0 arrays, followed by pathway analysis and qRT-PCR validation of selected genes. Relative to vehicle-control reference tissues, 173 genes were differentially expressed at week 6, whereas 1628 genes were differentially expressed at week 29, indicating marked expansion of transcriptional dysregulation during carcinogenic progression. Sixteen genes were differentially expressed only at week 6, while 157 genes were altered at both time points. Pathway analysis suggested that inflammatory and immunologic processes were prominent during the early response to NMBA exposure, whereas late-stage carcinogenesis was characterized by NRF2-associated oxidative-stress response and dysregulation of multiple glutathione S-transferase family members. A subset of progression-associated genes, including *Defb4*, *Gsta2*, *Sbsn*, *Spink5*, *Plcd4*, *Hbb*, and *Hba-a2*, showed increasing dysregulation from week 6 to week 29. These findings define temporally distinct molecular programs in NMBA-induced esophageal squamous carcinogenesis and provide a framework for prioritizing candidate pathways and genes relevant to esophageal cancer prevention, early detection, and progression biology.

## 1. Introduction

Esophageal cancer remains a major cause of cancer-related mortality worldwide, with esophageal squamous cell carcinoma (ESCC) representing the predominant histologic subtype globally [1,2,3]. Despite advances in endoscopic diagnosis, surgery, radiotherapy, chemotherapy, immunotherapy, and multimodality treatment, the overall prognosis for ESCC remains poor, largely because many patients are diagnosed at advanced stages [2,3]. These clinical challenges underscore the need to better understand the molecular events that occur during early esophageal carcinogenesis, and to identify biomarkers and pathways that may inform prevention, early detection, and mechanism-based intervention [4,5].

ESCC develops through a multistage process characterized by progressive histopathologic alterations, including normal squamous epithelium, basal cell hyperplasia, dysplasia, carcinoma in situ, and invasive ESCC [4,5,6]. The risk of progression increases with the severity of dysplasia, making premalignant lesions biologically and clinically important targets for cancer prevention [7,8,9]. However, systematic molecular characterization of early human ESCC precursor lesions remains challenging. In clinical practice, severe dysplasia and carcinoma in situ are more likely to undergo therapeutic intervention, whereas mild or moderate dysplasia may be monitored by surveillance endoscopy [5,10,11]. As a result, well-preserved human tissue samples representing sequential stages of early ESCC development are relatively limited. This limitation has hindered efforts to define the dynamic molecular changes that accompany the transition from early epithelial abnormalities to invasive carcinoma [12,13,14,15].

The *N*-nitrosomethylbenzylamine (NMBA)-induced Fischer-344 rat model is a well-established experimental system for studying esophageal squamous cell carcinogenesis and evaluating chemopreventive interventions [16,17,18,19,20,21,22]. NMBA is among the most potent nitrosamine carcinogens for the rat esophagus, and repeated subcutaneous administration induces a reproducible sequence of squamous epithelial alterations, including basal cell hyperplasia, low- and high-grade dysplasia, papilloma formation, and invasive squamous cell carcinoma. These preneoplastic and neoplastic alterations recapitulate key histopathologic features of squamous epithelial carcinogenesis and can be quantitatively assessed over time. A major advantage of this model is that esophageal tissues can be collected at defined intervals after carcinogen exposure, enabling controlled evaluation of molecular alterations during early, intermediate, and late phases of carcinogenesis [23]. Previous studies have shown that NMBA-treated rats develop progressive epithelial abnormalities before overt tumor formation, followed by high tumor incidence at later time points [16,23]. In the standard NMBA-induced rat esophageal carcinogenesis bioassay, subcutaneous administration of NMBA at 0.25 mg/kg body weight three times per week for 5 weeks induces early preneoplastic lesions, predominantly epithelial hyperplasia and low-grade dysplasia, by approximately week 6, followed by high tumor incidence by week 29 [16,23]. Therefore, this model provides a useful platform for identifying stage-associated molecular events and candidate biomarkers relevant to ESCC prevention and progression.

Cancer development is accompanied by coordinated remodeling of gene-expression programs that regulate epithelial proliferation and differentiation, inflammatory and immune signaling, oxidative-stress adaptation, metabolic reprogramming, and extracellular matrix remodeling [24,25,26,27,28]. Although genomic, transcriptomic, proteomic, and sequencing-based approaches have substantially advanced the molecular characterization of ESCC, many studies have focused primarily on established tumors or comparisons between tumors and adjacent nontumor tissues [13,29,30,31,32]. Such cross-sectional analyses are valuable but may not fully distinguish early molecular events involved in carcinogen response and early carcinogenic progression from later changes associated with tumor expansion, tissue remodeling, inflammation, oxidative stress, or adaptive survival. Studies comparing precursor lesions, tumor stages, or experimentally defined time points support the value of stage-resolved molecular profiling for understanding ESCC evolution [33,34,35]. Temporal transcriptomic profiling across defined stages of carcinogenesis can therefore provide a more dynamic view of ESCC development and may help identify early, prevention-relevant pathways as well as late, progression-associated molecular programs.

In the present study, we used the NMBA-induced rat model to investigate temporal gene-expression changes during esophageal squamous carcinogenesis. NMBA-treated esophageal tissues were collected at defined time points to represent distinct phases of carcinogenic progression, and genome-wide microarray analysis was performed to identify differentially regulated genes, biological functions, and canonical pathways associated with early and late stages of carcinogenesis. We hypothesized that NMBA-induced esophageal carcinogenesis is characterized by stage-associated molecular programs, with early carcinogen-induced inflammatory and immunologic responses followed by broader late-stage stress adaptation and oxidative-stress remodeling. Through this approach, we sought to identify candidate pathways and progression-associated genes that may inform future studies of ESCC prevention, early detection, and therapeutic intervention.

## 2. Results

### 2.1. NMBA Induces Stage-Dependent Transcriptional Remodeling

**Figure 1:**
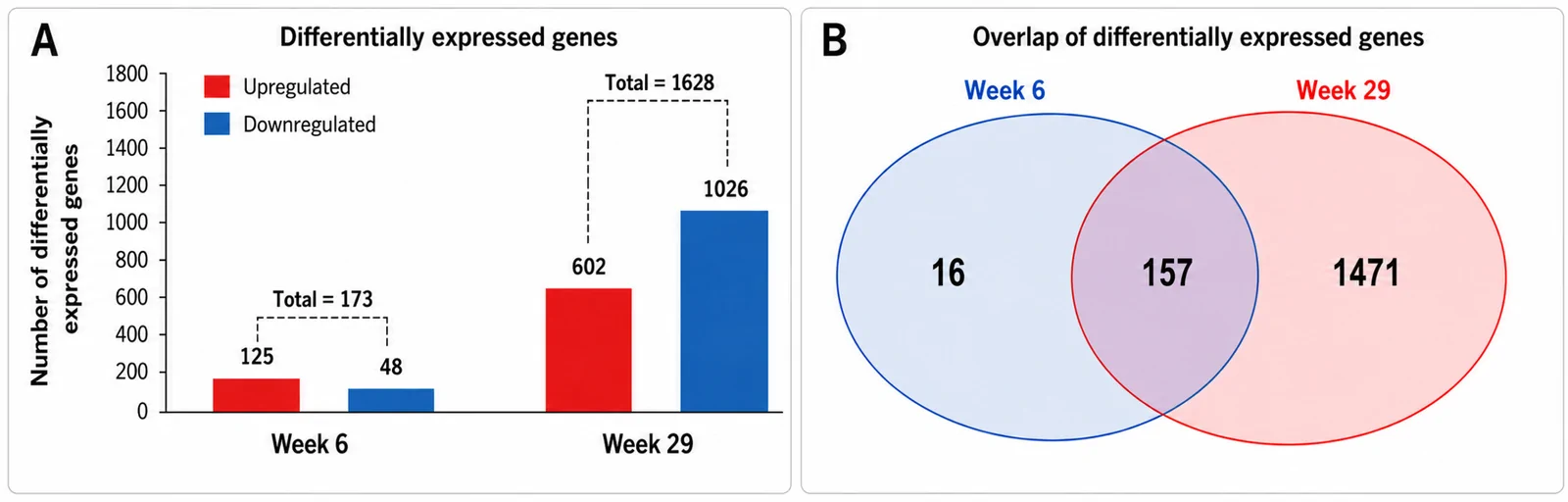
Temporal transcriptional changes during NMBA-induced esophageal squamous carcinogenesis. (**A**) Numbers of upregulated and downregulated differentially expressed genes at week 6 and week 29 after NMBA exposure. Genes were considered differentially expressed using a cutoff of *p* < 0.001 and an absolute fold change ≥ 2.0 relative to vehicle-control reference tissue. (**B**) Venn diagram showing differentially expressed genes unique to week 6, unique to week 29, or shared between the two time points.

To determine whether NMBA-induced esophageal squamous carcinogenesis is associated with stage-dependent transcriptional changes, differentially expressed genes were identified in NMBA-treated esophageal tissues collected at week 6 and week 29 relative to vehicle-control reference tissues. Relative to vehicle-control reference tissues, 173 genes were differentially expressed at week 6 after NMBA exposure, including 125 upregulated genes and 48 downregulated genes. In contrast, 1628 genes were differentially expressed at week 29, including 602 upregulated genes and 1026 downregulated genes (Figure 1A). Thus, the number of dysregulated genes increased markedly from week 6 to week 29, consistent with the progressive expansion of transcriptional remodeling during NMBA-induced esophageal carcinogenesis.

Venn diagram analysis further demonstrated distinct and overlapping gene-expression changes between the two time points. Sixteen genes were differentially expressed only at week 6, whereas 1471 genes were differentially expressed only at week 29. In addition, 157 genes were differentially expressed at both week 6 and week 29 (Figure 1B).

To further evaluate global expression patterns across the microarray samples, principal component analysis and sample correlation analysis were performed using normalized expression profiles from the nine pooled microarray samples. PCA showed the overall distribution of vehicle-control reference, NMBA week 6, and NMBA week 29 samples, whereas Pearson correlation analysis showed high sample-to-sample correlations across the analyzed arrays (Supplementary Figure S1).

### 2.2. Early NMBA Exposure Is Associated with a Restricted Early-Response Gene Set

Among the 173 genes differentially expressed at week 6, 16 genes were altered only at this early time point and were not differentially expressed at week 29. These week 6-specific genes included *Slfn3*, *Serpinb2*, *Igfbp3*, *RGD1563091*, *Lgals3bp*, *Id1*, *LOC682861*, *Cyp26b1*, *Gbp2*, *rCG_35099*, *Psmb9*, *Tes*, *Scarb1*, *Psmb8*, *Itga1*, and *Agpat4* (Table 1).

### 2.3. qRT-PCR Validates Selected Microarray Findings

qRT-PCR was performed for selected differentially expressed genes including *Lgals3bp*, *Psmb9*, *Serpinb2*, *Gbp2*, *Hba-a2*, *Igfbp3*, *Defb4*, *Plcd4*, and *Hbb*. These genes represent week 6-specific genes, genes altered at both time points, and candidate progression-associated genes.

**Figure 2:**
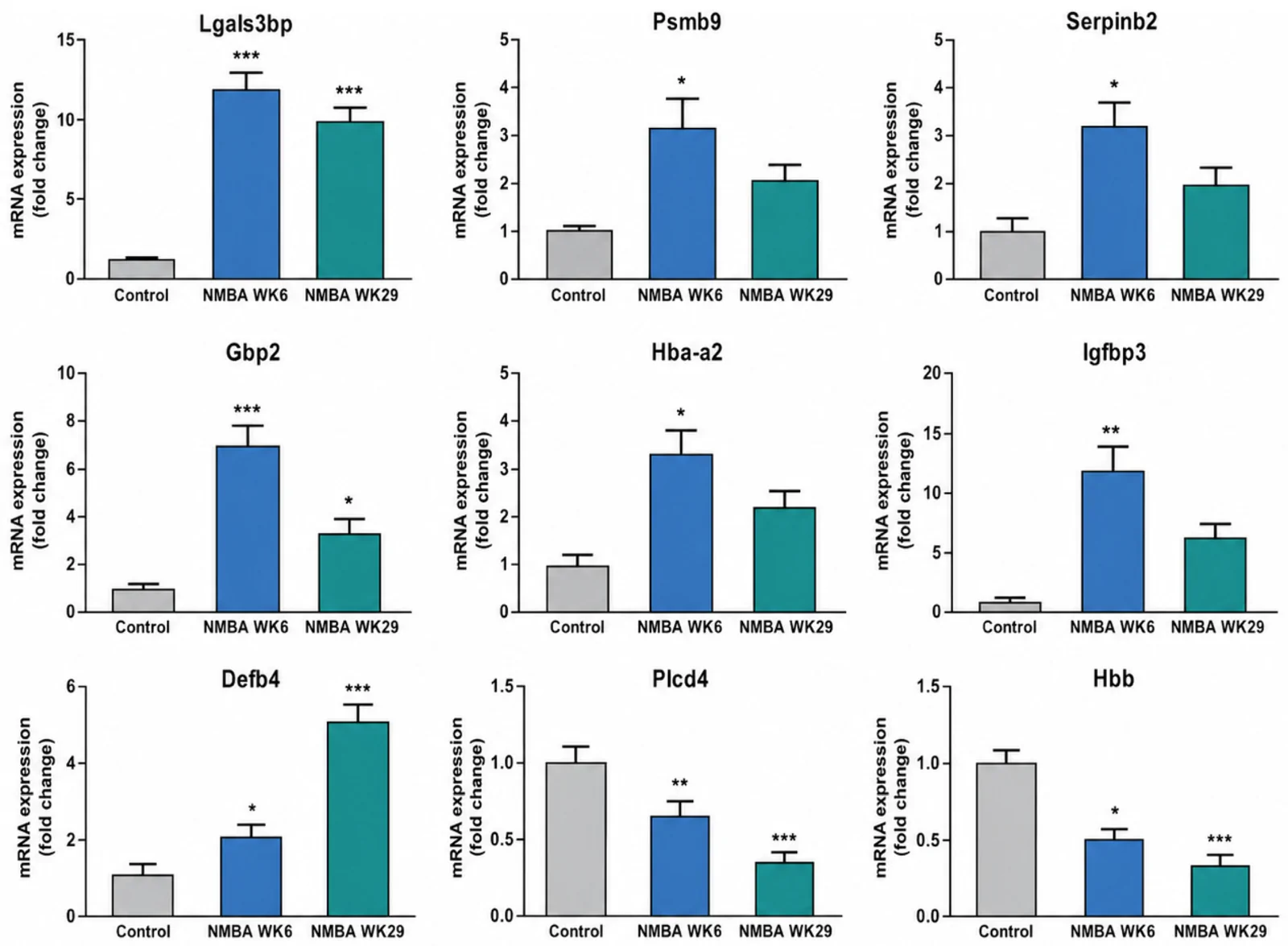
qRT-PCR validation of selected genes identified by microarray analysis during NMBA-induced esophageal squamous carcinogenesis. qRT-PCR validation was performed using 27 individual rat esophageal RNA samples, including nine vehicle-control samples, nine NMBA-treated week 6 samples, and nine NMBA-treated week 29 samples, corresponding to *n* = 9 biological replicates per group. Quantitative RT-PCR was performed to validate selected differentially expressed genes identified by microarray analysis. The selected genes included week 6-specific genes, genes altered at both time points, and candidate progression-associated genes. Relative mRNA expression is shown as fold change compared with vehicle-control tissues after normalization to *Gapdh* using the 2^−ΔΔCt^ method. Bars represent mean ± SD. Statistical significance was determined using one-way ANOVA followed by Dunnett’s multiple-comparison test comparing each NMBA-treated group with the vehicle-control group. * *p* < 0.05, ** *p* < 0.01, and *** *p* < 0.001 versus control.

The qRT-PCR results generally confirmed the expression patterns observed in the microarray analysis for the selected genes (Figure 2).

### 2.4. IPA Identifies Early Inflammatory/Immunologic Networks and Late Functional Enrichment

To identify biological functions and regulatory networks associated with NMBA-induced transcriptional changes, differentially expressed genes from weeks 6 and 29 were analyzed separately using Ingenuity Pathway Analysis. At week 6, the enriched disease categories were dominated by inflammatory disease and immunologic disease, involving 34 and 33 genes, respectively (Table 2). The top molecular and cellular functions at this early time point included cellular development, carbohydrate metabolism, cell death and survival, cellular growth and proliferation, and cellular function and maintenance. No canonical pathway reached the reporting threshold in the current IPA output at week 6.

For comparison, week 29 showed substantially broader functional enrichment, with cancer emerging as the dominant disease category and 446 associated genes (Table 2). Week 29 tissues were also enriched for cellular development, cell morphology, cell death and survival, carbohydrate metabolism, and molecular transport. Canonical pathway analysis at week 29 identified NRF2-associated oxidative-stress response, protein kinase A signaling, mitochondrial dysfunction, and AMPK signaling as significantly enriched pathways.

**Figure 3:**
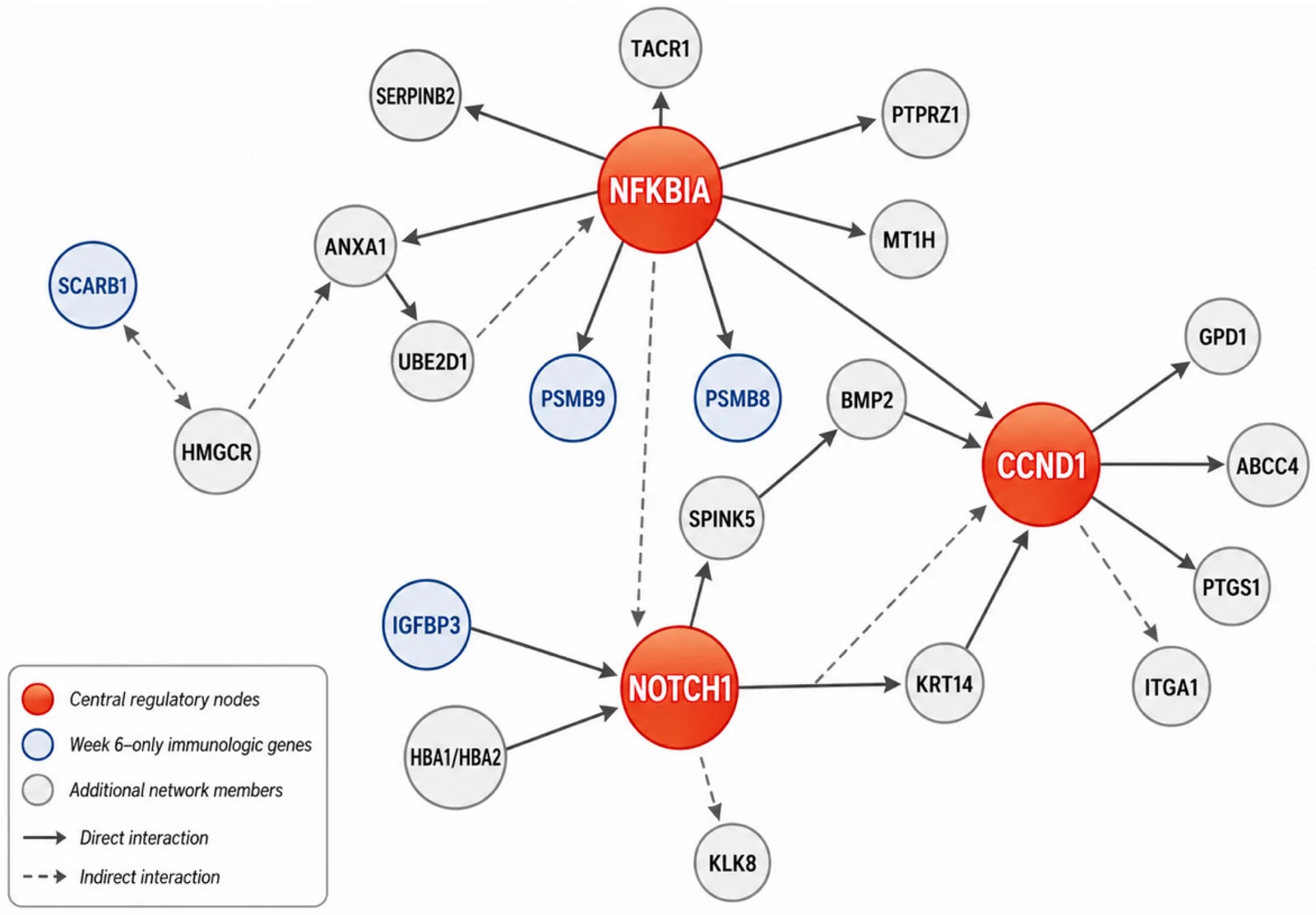
Early inflammatory/immunologic interaction network at week 6 during NMBA-induced esophageal squamous carcinogenesis. An interaction network was constructed using inflammation- and immunology-associated genes identified among the differentially expressed genes in NMBA-treated rat esophageal tissues at week 6 compared with vehicle-control reference tissues. The network highlights NFKBIA, NOTCH1, and CCND1 as central regulatory nodes within the early inflammatory/immunologic program. Selected genes that were differentially expressed only at week 6 and linked to immunologic functions are highlighted in blue, whereas additional connected network members are shown in gray. Solid arrows indicate direct interactions, and dashed arrows indicate indirect interactions.

The week 6 inflammation/immunology-associated interaction network identified NFKBIA, NOTCH1, and CCND1 as central regulatory nodes (Figure 3). Among the 16 genes that were differentially expressed only at week 6, *Psmb8*, *Psmb9*, *Igfbp3*, and *Scarb1* were associated with immunologic disease-related functions.

### 2.5. Late Carcinogenesis Shows Enrichment of NRF2-Associated Oxidative-Stress Response

In contrast to the restricted week-6-specific gene set, week 29 showed broad transcriptional dysregulation and enrichment of cancer-associated disease categories and canonical pathways. IPA analysis identified cancer as the dominant disease category at week 29, with 446 associated genes. The major molecular and cellular functions altered at week 29 included cellular development, cell morphology, cell death and survival, carbohydrate metabolism, and molecular transport. Canonical pathway analysis identified NRF2-associated oxidative-stress response, protein kinase A signaling, mitochondrial dysfunction, and AMP-activated protein kinase signaling as significantly enriched pathways (Table 2).

**Figure 4:**
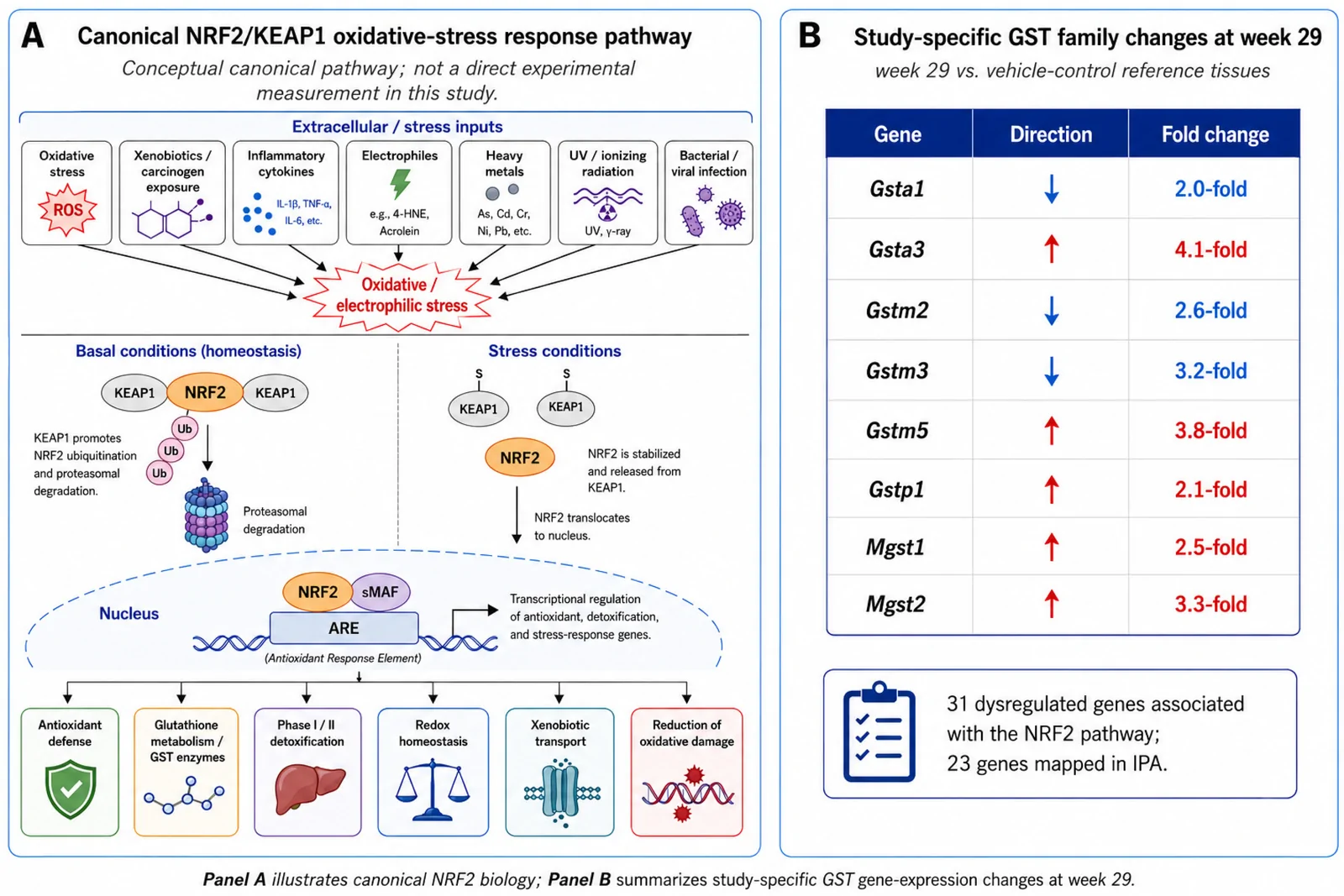
NRF2-associated oxidative-stress response pathway at week 29 during NMBA-induced esophageal squamous carcinogenesis. (**A**) Canonical NRF2/KEAP1 oxidative-stress response pathway. Under basal conditions, NRF2 is retained in the cytoplasm by KEAP1 and targeted for ubiquitination and proteasomal degradation. In response to oxidative or electrophilic stress, NRF2 is stabilized, translocates to the nucleus, dimerizes with small MAF proteins, and binds antioxidant response elements to regulate genes involved in antioxidant defense, detoxification, redox homeostasis, and xenobiotic transport. (**B**) Study-specific glutathione S-transferase family gene-expression changes at week 29 compared with vehicle-control reference tissues. Several GST family members were dysregulated at week 29, including downregulation of *Gsta1* (−2.0-fold), *Gstm2* (−2.6-fold), and *Gstm3* (−3.2-fold), as well as upregulation of *Gsta3* (4.1-fold), *Gstm5* (3.8-fold), *Gstp1* (2.1-fold), *Mgst1* (2.5-fold), and *Mgst2* (3.3-fold). A total of 31 dysregulated genes were associated with the NRF2 pathway, of which 23 were mapped to the NRF2 pathway in Ingenuity Pathway Analysis. Red upward arrows indicate upregulation, and blue downward arrows indicate downregulation. (**A**) illustrates canonical NRF2 biology; (**B**) summarizes study-specific GST gene-expression changes at week 29.

At week 29, 31 dysregulated genes were associated with the NRF2 pathway, of which 23 mapped to the NRF2 pathway in IPA (Figure 4). Several glutathione S-transferase family members were dysregulated at week 29, including downregulation of *Gsta1*, *Gstm2*, and *Gstm3*, and upregulation of *Gsta3*, *Gstm5*, *Gstp1*, *Mgst1*, and *Mgst2*.

### 2.6. Progression-Associated Genes Show Increasing Dysregulation from Week 6 to Week 29

**Figure 5:**
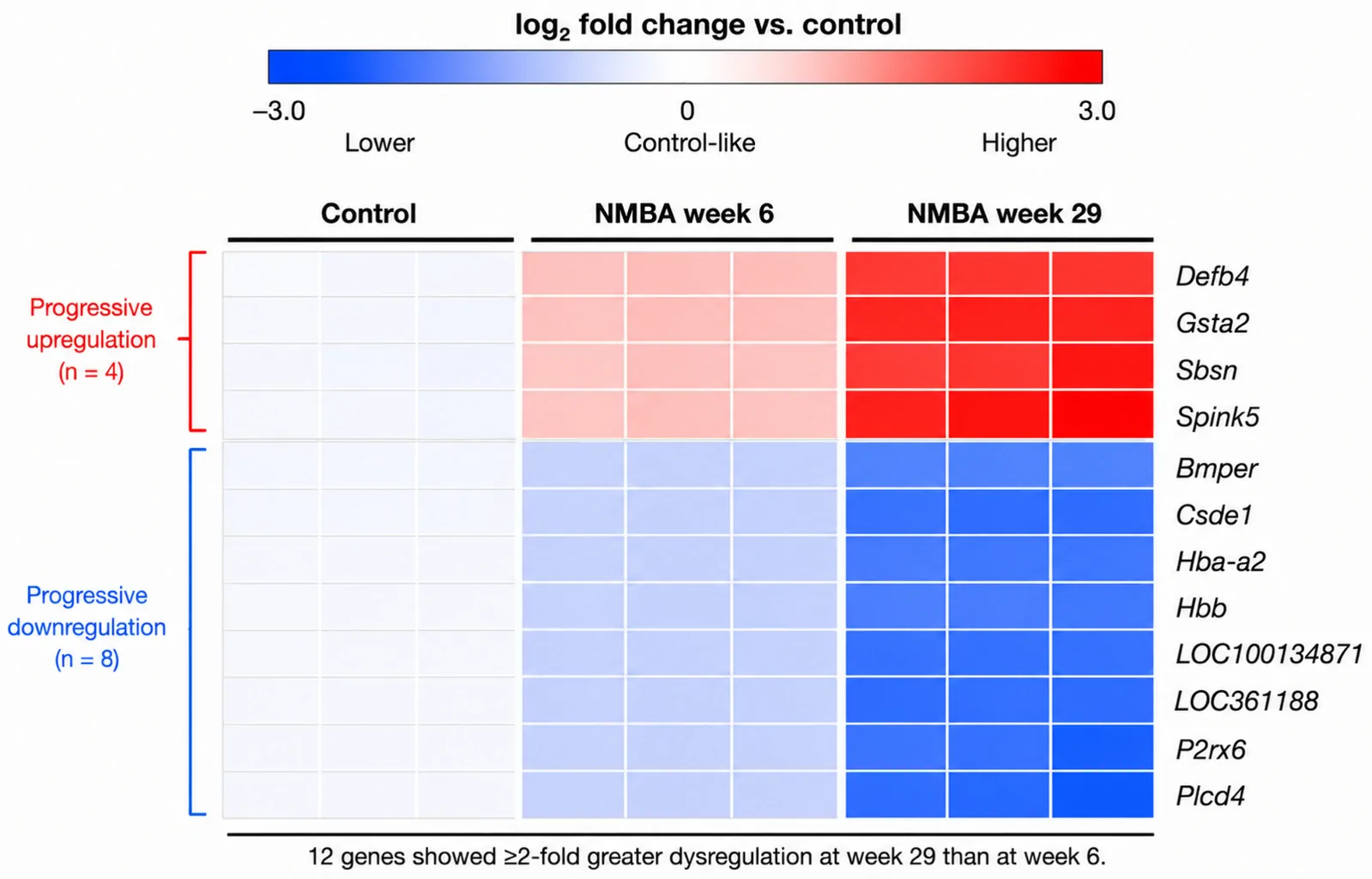
Progression-associated genes showing increasing dysregulation from week 6 to week 29 after NMBA exposure. Heatmap showing 12 genes that were differentially expressed at both week 6 and week 29 and exhibited at least a two-fold greater magnitude of dysregulation at week 29 compared with week 6. Four genes showed progressive upregulation (*Defb4*, *Gsta2*, *Sbsn*, and *Spink5*), whereas eight genes showed progressive downregulation (*Bmper*, *Csde1*, *Hba-a2*, *Hbb*, *LOC100134871*, *LOC361188*, *P2rx6*, and *Plcd4*). Colors indicate log_2_ fold change relative to vehicle-control reference tissues, with red indicating higher expression and blue indicating lower expression. Each column represents one pooled biological replicate generated by combining RNA from three individual rats. Three pooled biological replicates were analyzed for the vehicle-control group, three for the NMBA week 6 group, and three for the NMBA week 29 group.

Among the 157 genes differentially expressed at both week 6 and week 29, a subset showed increasing magnitude of dysregulation as carcinogenesis progressed. Twelve genes demonstrated at least a two-fold greater magnitude of dysregulation at week 29 compared to week 6: *Bmper*, *Csde1*, *Defb4*, *Gsta2*, *Hba-a2*, *Hbb*, *LOC100134871*, *LOC361188*, *P2rx6*, *Plcd4*, *Sbsn*, and *Spink5* (Table 3 and Figure 5).

Several of these genes showed progressive upregulation from week 6 to week 29. For example, *Defb4* increased from 2.53-fold at week 6 to 7.34-fold at week 29, *Gsta2* increased from 6.04-fold to 12.42-fold, *Sbsn* increased from 2.95-fold to 6.32-fold, and *Spink5* increased from 2.59-fold to 5.79-fold. Other genes showed progressively greater downregulation, including *Plcd4*, which changed from −2.50-fold at week 6 to −5.81-fold at week 29; *Hbb*, which changed from −2.31-fold to −5.01-fold; and *Hba-a2*, which changed from −2.15-fold to −4.35-fold.

## 3. Discussion

Esophageal squamous cell carcinogenesis is a multifactorial and multistep process shaped by carcinogen exposure, epithelial injury, inflammation, oxidative stress, altered differentiation, and progressive molecular remodeling. In the present study, we used the NMBA-induced rat model to characterize time-dependent gene-expression changes during esophageal squamous carcinogenesis. By comparing early and late time points after NMBA exposure, we identified stage-associated transcriptional programs that distinguish the early molecular response to carcinogen-induced epithelial injury from the broader molecular remodeling observed at later stages of disease development. The major finding of this study is that early NMBA-induced carcinogenesis was characterized by a relatively restricted gene-expression response enriched for inflammatory and immunologic functions, whereas late-stage carcinogenesis was associated with markedly expanded transcriptional dysregulation and prominent NRF2-associated oxidative-stress and detoxification pathway remodeling. In addition, we identified a subset of progression-associated genes whose magnitude of dysregulation increased from week 6 to week 29, providing candidate molecular markers for future studies of ESCC development, prevention, and progression. qRT-PCR validation of selected genes further supported the reliability of the microarray findings and confirmed reproducible NMBA-associated expression changes in representative genes during esophageal carcinogenesis.

The NMBA-induced rat model has been extensively used to study the molecular biology and chemoprevention of esophageal squamous carcinogenesis. Previous studies from our group and others have demonstrated that this model produces reproducible squamous epithelial abnormalities and is responsive to chemopreventive interventions, including black raspberries, phenylethyl isothiocyanate, and agents targeting inflammatory or carcinogenesis-associated pathways [16,17,18,36]. Earlier gene-expression studies in this model identified carcinogen-responsive genes and gene sets whose expression could be modulated toward normal levels by preventive agents [16,23,37]. These studies provided important insights into the molecular effects of chemoprevention; however, they were primarily focused on intervention-associated reversal of carcinogen-induced expression changes. In contrast, the present study was designed to examine time-dependent molecular changes during NMBA-induced esophageal carcinogenesis itself. This approach allowed us to compare early and late transcriptional programs and to identify molecular pathways more closely associated with early carcinogen response, carcinogenic progression, and later-stage tissue remodeling.

At week 6, NMBA exposure induced a relatively limited transcriptional response, with 173 differentially expressed genes compared with vehicle-control tissues. Pathway analysis suggested that inflammatory and immunologic processes were prominent features of this early phase. This finding is biologically plausible, as carcinogen-induced epithelial injury can trigger innate immune activation, cytokine signaling, proteasome-related antigen-processing pathways, epithelial stress responses, and early alterations in cell survival and proliferation. Network analysis identified NF-κB, Notch, and CCND1 as central nodes within the inflammation- and immunology-associated gene network, suggesting a potential convergence of inflammatory signaling, epithelial differentiation, and proliferative control during the early response to NMBA exposure. NF-κB is a central transcriptional mediator linking chronic inflammation to cancer promotion by regulating proinflammatory cytokines and chemokines, inflammatory enzymes such as COX-2 and iNOS, anti-apoptotic mediators, and cell-survival programs [38,39,40,41]. Notch signaling regulates squamous epithelial differentiation, stem/progenitor cell fate, and immune-cell interactions, and context-dependent disruption or activation of Notch signaling has been implicated in squamous epithelial neoplasia [42,43,44,45]. *CCND1* encodes cyclin D1, a key regulator of G1–S cell-cycle progression and epithelial proliferation. Its position within the early inflammatory network, together with prior evidence that NF-κB can transcriptionally regulate cyclin D1, supports a potential link between immune/inflammatory activation and proliferative expansion during NMBA-induced esophageal carcinogenesis [46,47,48,49].

Among the genes differentially expressed only at week 6, several have plausible links to immune regulation, inflammatory signaling, proteasome function, epithelial remodeling, or cellular stress responses. The limited number of week 6-specific genes, compared with the larger number of week 29-specific genes, supports the interpretation that early NMBA exposure is associated with a relatively restricted transcriptional response before the emergence of broad late-stage transcriptional dysregulation. For example, *Psmb8* and *Psmb9* encode proteasome-related components involved in protein turnover and antigen processing, whereas *Gbp2* is associated with interferon-responsive immune signaling [50,51,52]. Other early-restricted genes, including *Lgals3bp*, *Igfbp3*, *Scarb1*, and *Serpinb2*, have been implicated in inflammatory regulation, epithelial remodeling, immune response, or tumor-associated signaling [53,54,55,56]. Together with the enrichment of inflammatory and immunologic disease categories at week 6, these findings support the possibility that early NMBA exposure induces a focused transcriptional response related to epithelial injury and host-response pathways. Although the functional significance of these individual genes in NMBA-induced esophageal carcinogenesis remains to be determined, their restriction to the early time point suggests that they may reflect an early epithelial injury or host-response program induced by carcinogen exposure before the emergence of broad late-stage transcriptional dysregulation. These findings support the concept that early squamous carcinogenesis is not simply a lower-magnitude version of late-stage disease, but may involve distinct molecular processes related to tissue injury, immune activation, proteostasis, and epithelial adaptation.

By week 29, the number of differentially expressed genes increased substantially to 1628, indicating broad transcriptional remodeling during later stages of NMBA-induced esophageal carcinogenesis. IPA analysis identified enrichment of cancer-related functions and pathways associated with cellular development, cell morphology, cell death and survival, carbohydrate metabolism, molecular transport, mitochondrial dysfunction, AMP-activated protein kinase signaling, protein kinase A signaling, and NRF2-associated oxidative-stress response. In contrast to the more restricted inflammatory and immunologic enrichment observed at week 6, the week 29 findings indicate broader cancer-associated transcriptional remodeling during later stages of NMBA-induced esophageal squamous carcinogenesis. This expansion of dysregulated genes is consistent with progressive tissue remodeling, altered epithelial differentiation, metabolic adaptation, survival signaling, and activation of cellular stress-response programs during advancing carcinogenesis. Importantly, the week 29 transcriptome was not defined solely by proliferative or tumor-associated pathways, but also by strong representation of oxidative-stress and detoxification programs.

The enrichment of NRF2-mediated oxidative-stress response at week 29 is one of the most notable findings of this study. NRF2, encoded by *Nfe2l2*, is a master transcriptional regulator of antioxidant defense, xenobiotic metabolism, glutathione homeostasis, and cellular adaptation to electrophilic and oxidative stress [57,58,59,60]. In normal and premalignant tissues, NRF2 pathway activity can protect cells from carcinogen-induced damage by promoting detoxification and antioxidant-response pathways [60,61]. However, persistent NRF2 pathway engagement in established or progressing lesions may also support tumor-cell survival by enhancing redox buffering, metabolic flexibility, detoxification capacity, and resistance to cellular stress [59,61,62,63]. This dual role is particularly relevant to squamous cancers, including ESCC, in which alterations in the NRF2/KEAP1 pathway have been reported and may contribute to tumor progression, therapeutic resistance, or stress adaptation [64,65,66,67]. In the present study, multiple NRF2-associated genes were dysregulated at week 29, including several glutathione S-transferase family members. Altered expression of *Gsta1*, *Gsta3*, *Gstm2*, *Gstm3*, *Gstm5*, *Gstp1*, *Mgst1*, and *Mgst2* suggests substantial remodeling of detoxification and glutathione-dependent stress-response pathways during later stages of NMBA-induced carcinogenesis [68,69]. These findings support a model in which oxidative-stress adaptation becomes increasingly prominent as esophageal squamous lesions progress. Future studies incorporating protein-level validation of NRF2 pathway components will be important to determine whether the NRF2-associated transcriptional signature observed at week 29 reflects functional activation of NRF2-regulated antioxidant and detoxification pathways. Such studies should include assessment of NRF2 protein expression and subcellular localization, for example by nuclear and cytoplasmic fractionation followed by Western blot analysis using appropriate nuclear and cytoplasmic loading controls, as well as immunohistochemistry or immunofluorescence to evaluate NRF2 localization in esophageal tissues. Additional validation of NRF2 pathway components and downstream NRF2-associated proteins, including KEAP1, NQO1, and selected GST family proteins, would further strengthen the functional interpretation of this pathway.

The identification of 12 progression-associated genes whose magnitude of dysregulation increased from week 6 to week 29 provides an additional layer of biological insight into NMBA-induced esophageal carcinogenesis. Four genes, *Defb4*, *Gsta2*, *Sbsn*, and *Spink5*, showed progressive upregulation, whereas eight genes, *Bmper*, *Csde1*, *Hba-a2*, *Hbb*, *LOC100134871*, *LOC361188*, *P2rx6*, and *Plcd4*, showed progressive downregulation. These genes span several biological processes relevant to carcinogenesis, including epithelial barrier function, squamous differentiation, inflammatory defense, oxidative-stress response, RNA regulation, purinergic signaling, and oxygen transport-related pathways. *Defb4*, which encodes beta-defensin 4, is linked to epithelial antimicrobial defense and inflammatory/immune signaling and may reflect persistent epithelial immune activation [70,71]. *Gsta2* is consistent with the late enrichment of detoxification and oxidative-stress response pathways, as glutathione S-transferases are important components of cellular antioxidant and xenobiotic defense, and several *Gsta/Gstm* genes are regulated by NRF2-dependent pathways [68,72,73]. *Sbsn* and *Spink5* are associated with squamous epithelial differentiation and barrier-associated biology, suggesting that progressive epithelial remodeling is a feature of NMBA-induced carcinogenesis [74,75,76]. Conversely, progressive downregulation of *Plcd4*, *P2rx6*, *Csde1*, and hemoglobin-related transcripts may reflect disruption of phosphoinositide/calcium signaling, purinergic signaling, RNA regulation, epithelial homeostasis, oxygen-related biology, or changes in tissue composition during later stages. These genes should not yet be considered validated diagnostic or prognostic biomarkers; rather, they represent candidate progression-associated markers that warrant further validation in independent animal studies, human ESCC datasets, and premalignant or malignant esophageal tissues.

An important implication of this study is that time-dependent molecular profiling can provide information that is not readily captured by cross-sectional comparisons of established tumors and adjacent normal tissues. Human ESCC omics studies have substantially advanced understanding of genomic, transcriptomic, proteomic, and pathway alterations in established disease. However, tumor-normal comparisons often cannot distinguish molecular events involved in early carcinogen response or early carcinogenic progression from changes that arise later as consequences of tumor expansion, tissue remodeling, inflammation, oxidative stress, or adaptive survival. The NMBA model offers a complementary experimental approach because esophageal tissues can be collected at defined time points after carcinogen exposure. Although animal models cannot fully reproduce the complexity of human ESCC, stage-resolved profiling in a controlled carcinogenesis model can help prioritize pathways relevant to prevention, early detection, and stage-specific intervention. In this context, our findings suggest that inflammatory and immunologic pathways may be particularly important during the early response to NMBA exposure, whereas NRF2-associated oxidative-stress adaptation may become more prominent during later progression.

This study has several strengths, including the use of a well-characterized rat model of esophageal squamous carcinogenesis, comparison of defined early and late time points after carcinogen exposure, genome-wide expression profiling, pathway and network analyses, and qRT-PCR validation of selected genes. The time-dependent study design provides a framework for distinguishing early response programs from later progression-associated molecular alterations. Several considerations should be noted. First, the microarray analysis was performed using pooled RNA samples, which supports discovery-level profiling but limits assessment of inter-individual variability. Second, the vehicle-control reference samples used for microarray analysis were collected from vehicle-treated rats at weeks 12, 15, and 18, rather than at the same time points as the NMBA-treated week 6 and week 29 samples. Therefore, age-related or time-dependent background transcriptional changes cannot be fully excluded. However, the vehicle-control pooled samples showed high sample-to-sample correlation and clustered together in the normalized expression analysis, supporting the internal consistency of the vehicle-control reference group. Third, formal multiple-testing correction was not applied in the original microarray discovery analysis; therefore, the differentially expressed gene lists should be interpreted as candidate discovery findings supported by stringent nominal *p*-value and fold-change thresholds, pathway-level analysis, and qRT-PCR validation of selected genes. Fourth, because the study was performed using a microarray platform and pathway analysis was based on the IPA Knowledge Base available at the time of the original analysis, future studies using RNA-sequencing and updated transcript annotation would further refine the gene-expression signatures identified here. Fifth, although selected genes were validated by qRT-PCR, additional validation at the protein level and in independent animal samples will be important to establish the biological relevance of these candidate progression-associated genes.

Future studies should also evaluate the clinical relevance of the inflammatory, NRF2-associated oxidative-stress, and progression-associated gene signatures identified in this model using publicly available human esophageal cancer transcriptomic datasets, including The Cancer Genome Atlas Esophageal Carcinoma cohort (TCGA-ESCA) and other independent cohorts. Such analyses should stratify tumors by histologic subtype, particularly ESCC versus esophageal adenocarcinoma, and use cross-species ortholog mapping and pathway-level approaches to determine whether the transcriptional programs observed in the NMBA-induced rat model are conserved in human ESCC. Because TCGA-ESCA consists primarily of established human tumors rather than sequentially collected premalignant tissues, such analyses would be complementary rather than directly equivalent to the temporal carcinogenesis model used in the present study.

## 4. Materials and Methods

### 4.1. Animal Model and Tissue Collection

The animal study was conducted as part of a larger investigation of NMBA-induced esophageal squamous carcinogenesis. The animal study was approved by The Ohio State University Institutional Animal Care and Use Committee (IACUC; approval No. 2009A0054-R5). All animal procedures were conducted in accordance with institutional guidelines for the care and use of laboratory animals. Four-week-old male Fischer 344 rats were purchased from Harlan Sprague–Dawley (Indianapolis, IN, USA). After arrival, rats were housed under standard laboratory conditions and placed on an AIN-76A diet (Dyets Inc., Bethlehem, PA, USA) during a two-week acclimation period. Animals were then randomly assigned to vehicle-control or NMBA-treated groups. NMBA was purchased from Ash Stevens (Detroit, MI, USA), and dimethyl sulfoxide (DMSO) was purchased from Sigma Chemical Co. (St. Louis, MO, USA).

Rats in the NMBA-treated group received subcutaneous injections of NMBA at 0.25 mg/kg body weight in 20% DMSO/H_2_O, three times per week for five weeks. Vehicle-control rats received subcutaneous injections of 0.2 mL of 20% DMSO/H_2_O on the same schedule. Animals were euthanized by CO_2_ asphyxiation at defined time points after the initiation of NMBA or vehicle treatment. Based on established histopathologic progression in our previous NMBA model studies [16,23], esophageal tissues from NMBA-treated rats at weeks 6 and 29 were selected for gene-expression profiling to represent early and late time points of NMBA-induced esophageal squamous carcinogenesis, respectively. Vehicle-control esophageal tissues used for microarray analysis were collected from vehicle-treated rats at weeks 12, 15, and 18, and served as a common vehicle-control reference group for differential-expression comparisons with NMBA-treated tissues collected at week 6 and week 29.

At necropsy, the esophagus from each rat was excised, opened longitudinally, and examined grossly. Visible tumors greater than 0.5 mm in any single dimension were counted, mapped, and measured in three dimensions: length, width, and height. Tumor volume was calculated using the formula for a prolate spheroid: length × width × height × π/6. Tumors were then removed from the esophagus and snap-frozen separately in liquid nitrogen. The remaining esophageal epithelium was stripped from the submucosal and muscularis layers, snap-frozen in liquid nitrogen, and stored at −80 °C until RNA extraction.

**Figure 6:**
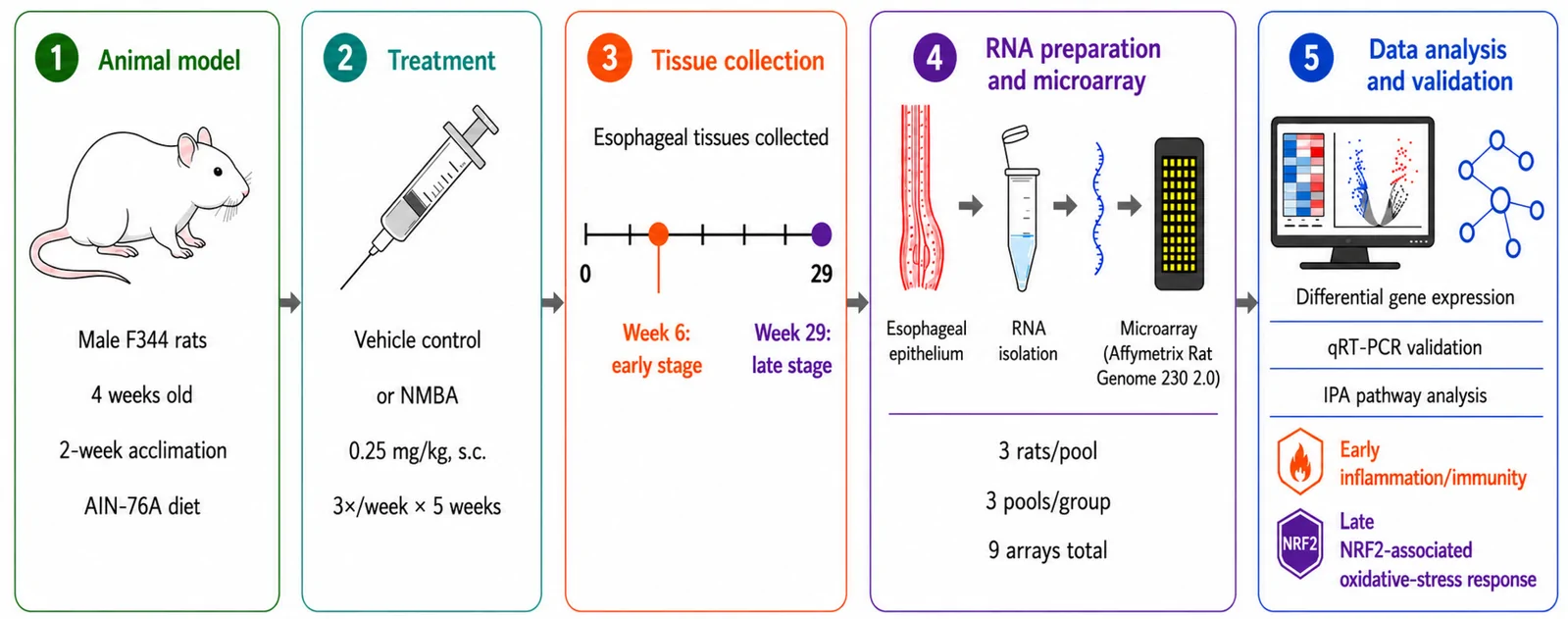
Experimental design for temporal transcriptomic profiling of NMBA-induced esophageal squamous carcinogenesis. Male F344 rats were acclimated for two weeks on an AIN-76A diet and then treated with vehicle or NMBA by subcutaneous injection three times per week for five weeks. NMBA-treated esophageal tissues collected at week 6 and week 29 were selected to represent early and late time points, respectively, based on prior histopathologic characterization of the NMBA model. Vehicle-control esophageal tissues for microarray analysis were collected from vehicle-treated rats at weeks 12, 15, and 18 and served as the vehicle-control reference group. RNA from three rats was pooled to generate one pooled biological replicate; three pooled biological replicates were analyzed for the vehicle-control group, three for the NMBA week 6 group, and three for the NMBA week 29 group using the Affymetrix Rat Genome 230 2.0 platform. Differential-expression analysis, qRT-PCR validation, and IPA pathway analysis were performed.

The experimental design and tissue processing workflow for temporal transcriptomic profiling are summarized in Figure 6.

### 4.2. RNA Isolation and Quality Control

Total RNA was isolated from frozen rat esophageal epithelial tissues using the RNeasy Mini Kit (Qiagen, Valencia, CA, USA) according to the manufacturer’s protocol. RNA integrity was assessed using an Agilent 2100 Bioanalyzer (Agilent Technologies, Santa Clara, CA, USA). Only RNA samples with an RNA integrity number (RIN) greater than 8 were used in the microarray analysis.

For transcriptomic profiling, three pooled vehicle-control RNA samples were generated from vehicle-treated rats collected at weeks 12, 15, and 18, respectively; each contained equal amounts of RNA from three individual rats. Three pooled RNA samples were generated from NMBA-treated rats collected at week 6, and three pooled RNA samples were generated from NMBA-treated rats collected at week 29. Thus, a total of nine pooled RNA samples, representing 27 individual rats, were used for microarray analysis.

### 4.3. Microarray Hybridization and Data Processing

Microarray hybridization and scanning were performed by the Ohio State University Comprehensive Cancer Center Microarray Facility. Gene-expression profiling was conducted using the Affymetrix Rat Genome 230 2.0 Array platform (Affymetrix, Santa Clara, CA, USA). A total of nine arrays were analyzed, including three pooled biological replicates from vehicle-control reference esophageal tissues, three pooled biological replicates from NMBA-treated esophageal tissues collected at week 6, and three pooled biological replicates from NMBA-treated esophageal tissues collected at week 29.

Raw signal intensities were processed using Affymetrix Expression Console software. Background correction, quantile normalization, and probe-level summarization were performed using the robust multi-array average (RMA) method [77]. Probe sets with low expression across arrays were filtered out using a noise-based filtering approach to remove transcripts with signal intensities below the detection threshold in most samples. Differential gene-expression analysis was performed using linear modeling with moderated t-statistics to improve variance estimation across the microarray data set [78]. Genes were considered differentially expressed if they met both of the following criteria: *p* < 0.001 and an absolute fold change of at least 2.0 compared with vehicle-control reference tissues.

### 4.4. qRT-PCR Validation

Selected differentially expressed genes identified by microarray analysis were validated by quantitative real-time PCR (qRT-PCR). RNA extraction and quality control procedures were performed as described above. cDNA was synthesized from total RNA using the High-Capacity cDNA Reverse Transcription Kit (Applied Biosystems, Foster City, CA, USA) according to the manufacturer’s protocol. For qRT-PCR validation, 27 individual rat esophageal RNA samples were analyzed, including nine vehicle-control samples, nine NMBA-treated week 6 samples, and nine NMBA-treated week 29 samples, corresponding to *n* = 9 biological replicates per group.

Genes were selected for qRT-PCR validation to represent distinct categories of the microarray findings, including week 6-specific genes, genes altered at both time points, and candidate progression-associated genes showing increased magnitude of dysregulation from week 6 to week 29. The selected genes were not chosen solely on the basis of rank order or fold-change magnitude but were selected based on a combination of differential-expression pattern, fold-change magnitude, biological relevance, and representation of key biological processes identified in the study, including inflammatory/immune signaling, proteasome-related response, epithelial remodeling, oxidative-stress response, and carcinogenic progression.

Gene expression was measured using TaqMan Gene Expression Assays and TaqMan Fast Advanced Master Mix (Applied Biosystems). The following TaqMan assays were used: *Lgals3bp* (Rn00478303_m1), *Psmb9* (Rn00562296_m1), *Serpinb2* (Rn00596662_m1), *Gbp2* (Rn01748315_m1), *Defb4* (Rn02532184_g1), *Plcd4* (Rn01401096_m1), *Hbb* (Rn00583657_g1), and *Hba-a2* (Rn01789798_s1). *Gapdh* (Rn01775763_g1) was used as the endogenous reference gene. *Gapdh* was selected based on prior internal validation and its established use in our laboratory for qRT-PCR analyses of rat esophageal tissues in the NMBA-induced carcinogenesis model. Relative gene-expression levels were calculated using the 2^−ΔΔCT^ method, with *Gapdh* as the endogenous reference gene and vehicle-control samples as the calibrator group.

Each sample was analyzed in technical triplicate using an ABI PRISM 7900 Sequence Detection System (Applied Biosystems). qRT-PCR data are presented as mean ± SD. Statistical differences in qRT-PCR expression levels among vehicle-control, NMBA week 6, and NMBA week 29 groups were assessed using one-way ANOVA followed by Dunnett’s multiple-comparison test, with each NMBA-treated group compared with the vehicle-control group.

### 4.5. IPA Pathway Analysis

Ingenuity Pathway Analysis (IPA; QIAGEN Inc., Redwood City, CA, USA) was used to identify biological functions, canonical pathways, and gene-interaction networks associated with NMBA-induced gene-expression changes. The IPA analyses were performed in October 2012 using IPA build version 172788 and Ingenuity Knowledge Base content version 14197757, release date 11 August 2012. The reference set was the Ingenuity Knowledge Base (Genes Only). Both direct and indirect relationships were included, and the analyses were filtered to include experimentally observed relationships. Differentially expressed genes from week 6 and week 29 were analyzed separately to identify time point-specific molecular pathways during esophageal squamous carcinogenesis.

For each analysis, the list of differentially expressed genes, corresponding gene identifiers, fold-change values, and statistical significance values were uploaded into IPA. Genes were mapped to curated molecular interaction, functional annotation, and canonical pathway databases within the Ingenuity Knowledge Base. IPA Core Analysis was performed to identify enriched biological functions, disease categories, canonical pathways, and molecular interaction networks. Enrichment of biological functions and pathways was assessed using IPA-generated statistical scores based on the overlap between the uploaded gene list and curated pathway or functional gene sets.

IPA network visualization was used to identify central regulatory nodes and interacting genes within selected functional networks. Pathway and network results were interpreted as hypothesis-generating, and they were used to prioritize biological processes and candidate genes for further mechanistic and translational evaluation.

### 4.6. Statistical Analysis

For microarray analysis, differentially expressed genes were identified by comparing NMBA-treated esophageal tissues at week 6 or week 29 with vehicle-control reference tissues. Linear modeling with moderated t-statistics was used to estimate differential expression across groups. For microarray differential-expression analysis, genes were considered differentially expressed if they met both a stringent nominal statistical threshold of *p* < 0.001 and an absolute fold-change cutoff of ≥2.0, compared with vehicle-control reference tissues. No formal multiple-testing correction was applied in the original discovery analysis; therefore, the differentially expressed gene lists were interpreted as discovery-level findings for pathway analysis and candidate-gene prioritization.

For qRT-PCR validation, relative gene-expression values were normalized to *Gapdh* and summarized as mean ± SD. Statistical differences among vehicle-control, NMBA week 6, and NMBA week 29 groups were evaluated using one-way ANOVA followed by Dunnett’s multiple-comparison test, with each NMBA-treated group compared with the vehicle-control group. A two-sided *p*-value < 0.05 was considered statistically significant for qRT-PCR analyses.

For IPA pathway analysis, the enrichment of biological functions, canonical pathways, and gene networks was assessed using IPA-generated statistical measures based on the overlap between the uploaded differentially expressed gene lists and curated gene sets in the Ingenuity Knowledge Base. IPA results were interpreted as hypothesis-generating pathway annotations rather than direct measures of pathway activation.

Principal component analysis and sample correlation analysis were performed using normalized microarray expression profiles. PCA was used to visualize global sample-level expression patterns across vehicle-control, NMBA week 6, and NMBA week 29 samples. Pearson correlation coefficients were calculated to assess sample-to-sample similarity, and hierarchical clustering was used to visualize relationships among the analyzed arrays.

## 5. Conclusions

This study demonstrates that NMBA-induced esophageal squamous carcinogenesis is associated with stage-associated transcriptional programs. Early after NMBA exposure, the esophageal epithelium exhibited a relatively restricted molecular response enriched for inflammatory and immunologic signaling, whereas the later stage of carcinogenesis was characterized by broad transcriptional dysregulation and prominent NRF2-associated oxidative-stress and detoxification pathway remodeling. In addition, a subset of progression-associated genes showed increasing dysregulation from week 6 to week 29, providing candidate molecular markers for future mechanistic and translational validation. Together, these findings support a time-dependent model of esophageal squamous carcinogenesis in which early inflammatory and immune activation is followed by later oxidative-stress adaptation programs. These results may help prioritize pathways and candidate genes for future studies of ESCC prevention, early detection, and progression biology.

## Figures and Tables

**Table 1:** Genes that are differentially expressed only at week 6 during NMBA-induced esophageal squamous carcinogenesis.

Gene Symbol	Gene Title	Fold Change
*Slfn3*	schlafen 3	3.85
*Serpinb2*	serine (or cysteine) peptidase inhibitor, clade B, member 2	2.81
*Igfbp3*	insulin-like growth factor binding protein 3	3.05
*RGD1563091*	similar to OEF2	2.27
*Lgals3bp*	lectin, galactoside-binding, soluble, 3 binding protein	2.17
*Id1*	inhibitor of DNA binding 1	2.33
*LOC682861*	similar to adenomatosis polyposis coli down-regulated 1	2.02
*Cyp26b1*	cytochrome P450, family 26, subfamily b, polypeptide 1	2.00
*Gbp2*	guanylate binding protein 2	2.37
*rCG_35099*	spinster homolog 2	2.10
*Psmb9*	proteasome (prosome, macropain) subunit, beta type 9 (large multifunctional peptidase 2)	2.31
*Tes*	testis derived transcript	2.15
*Scarb1*	scavenger receptor class B, member 1	2.17
*Psmb8*	proteasome (prosome, macropain) subunit, beta type 8 (large multifunctional peptidase 7)	2.02
*Itga1*	integrin alpha 1	2.23
*Agpat4*	1-acylglycerol-3-phosphate O-acyltransferase 4 (lysophosphatidic acyltransferase, delta)	2.20

**Table 2:** Enriched biological functions and canonical pathways associated with early and late NMBA-induced esophageal carcinogenesis.

IPA Category	Week 6 vs. Control	Week 29 vs. Control
Top network	Cancer, cellular development, cellular assembly and organization (20 genes)	Not available in current IPA output
Diseases and disorders	Inflammatory disease (34 genes; *p* = 3.12 × 10^−6^) Immunological disease (33 genes; *p* = 1.32 × 10^−5^)	Cancer (446 genes; *p* = 1.95 × 10^−15^)
Molecular and cellular functions	Cellular development (52 genes; *p* = 6.73 × 10^−7^) Carbohydrate metabolism (19 genes; *p* = 1.21 × 10^−5^) Cell death and survival (51 genes; *p* = 4.99 × 10^−5^) Cellular growth and proliferation (50 genes; *p* = 2.84 × 10^−5^) Cellular function and maintenance (30 genes; *p* = 8.29 × 10^−4^)	Cellular development (264 genes; *p* = 4.99 × 10^−10^) Cell morphology (235 genes; *p* = 3.06 × 10^−9^) Cell death and survival (324 genes; *p* = 5.63 × 10^−7^) Carbohydrate metabolism (116 genes; *p* = 8.94 × 10^−7^) Molecular transport (187 genes; *p* = 1.34 × 10^−5^)
Canonical pathways	No canonical pathway reached the reporting threshold in the current IPA output	NRF2-mediated oxidative-stress response (*p* = 9.90 × 10^−7^) Protein kinase A signaling (*p* = 1.15 × 10^−4^) Mitochondrial dysfunction (*p* = 4.90 × 10^−4^) AMPK signaling (*p* = 1.98 × 10^−4^)

Abbreviation: IPA, Ingenuity Pathway Analysis.

**Table 3:** Genes showing an increased magnitude of dysregulation from week 6 to week 29 during NMBA-induced esophageal squamous carcinogenesis.

Gene Symbol	Gene Title	Fold Change		
		Week 6 vs. Control	Week 29 vs. Control	Week 29/ Week 6
*Bmper*	BMP-binding endothelial regulator	−2.12	−4.80	2.27
*Csde1*	cold shock domain containing E1, RNA binding	−2.95	−6.69	2.27
*Defb4*	defensin beta 4	2.53	7.34	2.90
*Gsta2*	glutathione S-transferase A2 /// LOC494499 protein	6.04	12.42	2.06
*Hba-a2*	hemoglobin alpha, adult chain 2 /// hemoglobin alpha 2 chain	−2.15	−4.35	2.03
*Hbb*	hemoglobin, beta	−2.31	−5.01	2.17
*LOC100134871*	beta globin minor gene /// beta-globin	−2.53	−5.64	2.23
*LOC361188*	similar to WD repeat domain 17 /// similar to WD repeat domain 17	−2.30	−5.19	2.25
*P2rx6*	purinergic receptor P2X, ligand-gated ion channel, 6	−2.50	−5.02	2.01
*Plcd4*	phospholipase C, delta 4	−2.50	−5.81	2.33
*Sbsn*	suprabasin	2.95	6.32	2.14
*Spink5*	serine peptidase inhibitor, Kazal type 5	2.59	5.79	2.24

The genes listed showed at least a 2-fold greater magnitude of dysregulation at week 29 than at week 6.

## Data Availability

The original contributions presented in this study are included in the article. Further inquiries can be directed to the corresponding author.

## Supplementary Materials

The following supporting information can be downloaded at: https://www.mdpi.com/article/10.3390/ijms27177632/s1.

## Abbreviations

The following abbreviations are used in this manuscript:

AMPK	AMP-activated protein kinase
ARE	Antioxidant response element
CCND1	Cyclin D1
cDNA	Complementary DNA
DEG	Differentially expressed gene
DMSO	Dimethyl sulfoxide
ESCC	Esophageal squamous cell carcinoma
GEO	Gene Expression Omnibus
GST	Glutathione S-transferase
IPA	Ingenuity Pathway Analysis
KEAP1	Kelch-like ECH-associated protein 1
MAF	Musculoaponeurotic fibrosarcoma protein
NMBA	*N*-nitrosomethylbenzylamine
NRF2	Nuclear factor erythroid 2–related factor 2
PKA	Protein kinase A
qRT-PCR	Quantitative reverse-transcription polymerase chain reaction
RIN	RNA integrity number
RMA	Robust multi-array average
