# Supplementary material for: Temporal Transcriptomic Profiling of NMBA-Induced Rat Esophageal Squamous Carcinogenesis Identifies Early Inflammatory Activation and Late NRF2-Associated Oxidative-Stress Remodeling
Source: Int J Mol Sci. 2026 Aug 26;27(17):7632. doi: 10.3390/ijms27177632 (PMC13566898; doi:10.3390/ijms27177632)

Supplementary Figure S1. Principal component analysis and sample correlation of normalized microarray expression profiles.

Principal component analysis and Pearson correlation analysis were performed using normalized Affymetrix Rat Genome 230 2.0 expression profiles from the nine pooled microarray samples analyzed in this study. The analysis included three vehicle-control reference pooled samples generated from vehicle-treated rats collected at weeks 12, 15, and 18; three NMBA-treated week 6 pooled samples; and three NMBA-treated week 29 pooled samples. Each pooled sample was generated by combining equal amounts of RNA from three individual rats. Colors and symbols indicate experimental groups: vehicle control reference, blue circles; NMBA week 6, orange squares; and NMBA week 29, green triangles. (A) Principal component analysis showing overall sample-level expression patterns. Axes indicate the percentage of variance explained by PC1 and PC2, and shaded regions indicate group convex hulls. (B) Pearson correlation heatmap with unsupervised hierarchical clustering showing sample-to-sample similarity across the analyzed arrays. Numbers within cells indicate pairwise Pearson correlation coefficients, and the sample order reflects hierarchical clustering.

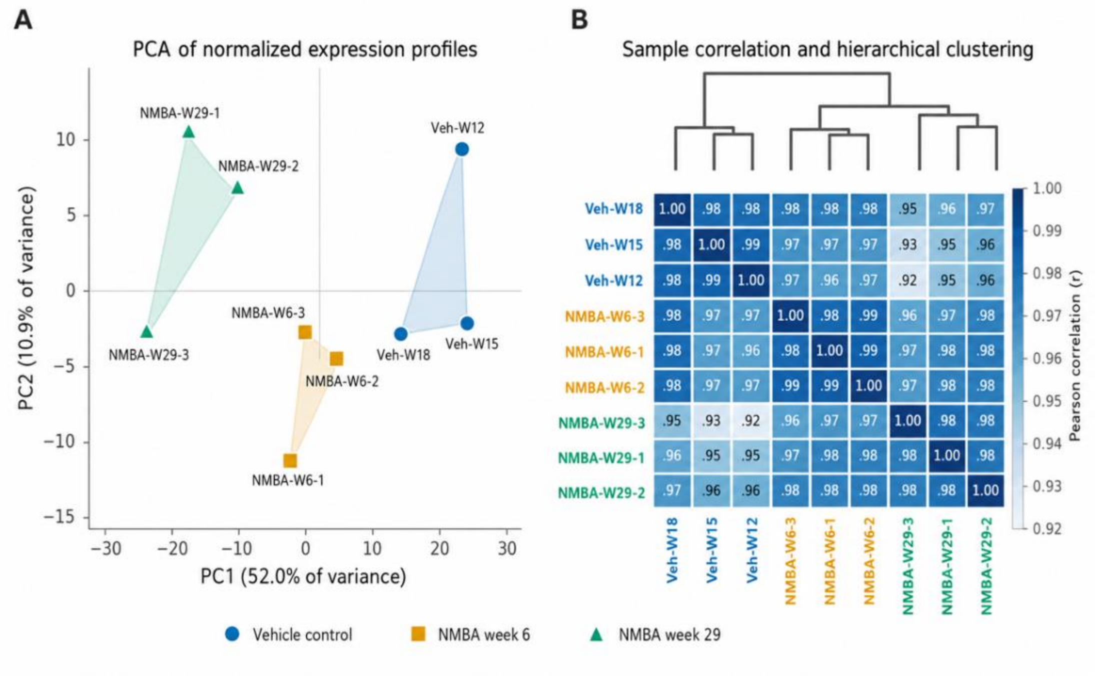

Supplement: Supplementary file 1 [file ijms-27-07632-s001.zip › ijms-4423321-supplementary.pdf]
